# Developmental Maturation of Dynamic Causal Control Signals in Higher-Order Cognition: A Neurocognitive Network Model

**DOI:** 10.1371/journal.pcbi.1002374

**Published:** 2012-02-02

**Authors:** Kaustubh Supekar, Vinod Menon

**Affiliations:** 1 Department of Psychiatry & Behavioral Sciences, Stanford University School of Medicine, Stanford, California, United States of America; 2 Department of Neurology & Neurological Sciences, Stanford University School of Medicine, Stanford, California, United States of America; 3 Program in Neuroscience, Stanford University School of Medicine, Stanford, California, United States of America; Indiana University, United States of America

## Abstract

Cognitive skills undergo protracted developmental changes resulting in proficiencies that are a hallmark of human cognition. One skill that develops over time is the ability to problem solve, which in turn relies on cognitive control and attention abilities. Here we use a novel multimodal neurocognitive network-based approach combining task-related fMRI, resting-state fMRI and diffusion tensor imaging (DTI) to investigate the maturation of control processes underlying problem solving skills in 7–9 year-old children. Our analysis focused on two key neurocognitive networks implicated in a wide range of cognitive tasks including control: the insula-cingulate salience network, anchored in anterior insula (AI), ventrolateral prefrontal cortex and anterior cingulate cortex, and the fronto-parietal central executive network, anchored in dorsolateral prefrontal cortex and posterior parietal cortex (PPC). We found that, by age 9, the AI node of the salience network is a major causal hub initiating control signals during problem solving. Critically, despite stronger AI activation, the strength of causal regulatory influences from AI to the PPC node of the central executive network was significantly weaker and contributed to lower levels of behavioral performance in children compared to adults. These results were validated using two different analytic methods for estimating causal interactions in fMRI data. In parallel, DTI-based tractography revealed weaker AI-PPC structural connectivity in children. Our findings point to a crucial role of AI connectivity, and its causal cross-network influences, in the maturation of dynamic top-down control signals underlying cognitive development. Overall, our study demonstrates how a unified neurocognitive network model when combined with multimodal imaging enhances our ability to generalize beyond individual task-activated foci and provides a common framework for elucidating key features of brain and cognitive development. The quantitative approach developed is likely to be useful in investigating neurodevelopmental disorders, in which control processes are impaired, such as autism and ADHD.

## Author Summary

The human brain undergoes significant maturational changes between childhood and adulthood that are thought to enable increasingly sophisticated thoughts and behaviors. One of the skills that we develop over time is the ability to problem solve, which relies in turn on the ability to control our attention and successfully direct our cognitive efforts. Using a novel multi-pronged neuroimaging approach, we identify for the first time the dynamic brain systems underlying the maturation of problem solving abilities. We find that the anterior insula, part of a larger network of regions previously shown to be important for salience processing and generating influential control signals, shows weaker influences over other key brain regions important for task performance in children compared to adults. In addition, structural connections between the anterior insula and other key regions were found to be weaker in children compared to adults. Importantly, measures of causal influences between key regions could be used to predict individual differences in behavioral performance. Our study is the first to show that the anterior insula, by virtue of its dynamic influences on other key brain regions, shows developmental differences in both structural and functional connectivity, which may contribute to more mature cognitive abilities in adulthood compared to childhood.

## Introduction

The development of increasingly sophisticated cognitive skills relies on the maturation of control processes for orienting attention and allocating resources for task relevant information [1], [2]. Such control processes are important for virtually every complex cognitive task, and there is growing evidence that they rely on functional interactions between multiple brain regions [3]. Despite the critical role of control processes in cognitive development, little is known about the maturation of functional brain systems underlying control mechanisms in the developing brain. Here we use a novel neurocognitive network approach with multimodal imaging to investigate the maturation of functional brain systems underlying control processes that support problem solving skills in young children.

Based on experimental studies across a wide range of cognitive domains, a number of cortical areas within the frontal lobe, including the anterior cingulate cortex (ACC), ventrolateral prefrontal cortex (VLPFC), dorsolateral prefrontal cortex (DLPFC) and the fronto-insular cortex (FIC) have emerged as putative sites for implementing different aspects of control [4],[5],[6],[7],[8],[9],[10]. Yet, even in adults, how these brain regions interact and implement control is poorly understood. This is especially surprising because, almost by definition, control processes should involve multiple interacting nodes of a network. A key challenge in untangling the potentially complex hierarchy of frontal control mechanisms is identifying patterns of their interconnectivity and how causal interactions emerge during performance of a cognitively demanding task. To date, however, there have been few systematic investigations of network interactions underlying control processes in adults and almost nothing is known about how these processes mature with development.

In this study we use a theoretically motivated approach to this problem based on neurocognitive network models derived from studies of intrinsic brain connectivity. Studies in adults have shown that the human brain is intrinsically organized into distinct functional networks [11], [12], [13]. Remarkably, intrinsic functional connectivity analysis has identified two distinct neurocognitive networks which are particularly important for implementing dynamic control across a wide range of cognitive tasks: a ‘salience network’ (SN) [12], anchored in the FIC and dorsal ACC, and a dorsal fronto-parietal ‘central executive network’ (CEN) anchored in the DLPFC and the supramarginal gyrus within the posterior parietal cortex (PPC) [4], [12], [14]. In adults the FIC node of the SN has been shown to play a major role in attentional capture, task-switching and generation of control signals that facilitate access to working memory resources necessary for a wide range of cognitive tasks [8]. The FIC consists of at least two cytoarchtectonically distinct regions – the VLPFC and the anterior insula (AI). While the VLPFC has been the focus of many investigations of control [15], [16], [17], there is growing evidence to suggest that the AI, by virtue of its tight coupling with the ACC, plays a critical and distinctive role [8], [14], [18], [19]. Notably, analysis of dynamic causal interactions has suggested that the AI initiates control signals which engage the ACC, DLPFC and PPC while disengaging the default mode network during cognitively challenging tasks [8]. In this study we use a neurocognitive network model based on the SN and CEN for investigating fundamental mechanisms mediating the development of dynamic control processes during cognition.

Over the past decade, several studies have examined developmental changes in the recruitment of brain areas belonging to these networks using cognitive tasks ranging from response inhibition, attention, and memory, to decision-making, reasoning and problem solving [20], [21], [22], [23], [24], [25], [26], [27]. Both increased and decreased recruitment of insula-cingulate and fronto-parietal systems have been reported over the course of development [28]. Although developmental neuroimaging studies have provided evidence for immature task-related activation in the VLPFC, AI, ACC, and DLPFC [1], [20], [25], [28], nothing is currently known about the maturation of dynamic interactions between these brain regions. Based on previous studies which have pointed to developmental changes in activation of areas that overlap with the SN and CEN we hypothesized that a neurocognitive network model would help clarify and significantly enhance our understanding of the mechanisms by which control processes mature in children.

A systematic network approach has the potential for providing insights into general development mechanisms mediating dynamic control processes during cognition. However, in both adults and children, the differential role and primacy of control signals has been difficult to disentangle, partly because these areas are typically coactivated during a wide range of cognitive tasks [12]. More specifically, it has been difficult to disambiguate the contributions of multiple overlapping frontal lobe regions using task-based functional magnetic resonance imaging (fMRI). Critically, the SN and CEN are often co-activated during cognitive tasks in children and adults, and isolating focal responses in a consistent manner from task-based fMRI activations is not straightforward. This is especially true in developmental studies since children tend to show more diffuse activations in the prefrontal cortex, making it difficult to disambiguate regional functional cortical responses [29]. To address this issue in a principled manner, we used multimodal imaging combining resting-state fMRI, cognitive task fMRI and DTI to examine developmental changes in dynamic interactions between the SN and CEN during cognition, and the underlying structural connectivity. Resting-state fMRI (rsfMRI) data were acquired and used to characterize the SN and CEN and to identify their five major nodes (SN: AI, VLPFC, ACC and CEN: DLPFC, PPC). We demarcated SN and CEN, and their nodes using analysis of rsfMRI data. An arithmetic problem solving task was used to investigate dynamic interactions between the SN and CEN during cognition. The arithmetic task used is easily understood and performed with high levels of accuracy by most 7–9 year old children, and several previous imaging studies have shown that it consistently activates all major nodes of the SN and CEN in both children and adults [28], [30]. DTI, performed in the same group of children and adults, was used to examine whether maturation of functional interactions between specific brain regions was related to the maturation of white matter pathways that link them. We predicted that the AI node of the SN would be a hub mediating dynamic causal interactions in adults but not in children. We further predicted that, compared to adults, children would have weaker dynamic causal interactions between the SN and CEN, and that weaker causal interactions would contribute significantly to reduced levels of activation as well as lower levels of task performance in children. Linking functional and structural connectivity measures, we predicted that immature causal interactions in children would be reflected in weaker integrity and density of white matter pathways linking key nodes of the SN and CEN. Together, these findings would provide novel information on temporal hierarchy of among prefrontal and parietal regions implicated in control processes [5], [16], [17] and for immature fronto-parietal causal control signals in children.

## Results

### Behavior

Children (ages 7–9) and adults (19–22) did not differ on IQ (*p* = 0.93) or gender (*p* = 0.75) (Table S1). Although both groups performed the arithmetic problem solving task with a high level of accuracy, children were significantly less accurate (*t* (38) = 5.54; *p*<0.001) and slower (*t(38)* = 10.99; *p*<0.001) than adults (Figure S1).

### Identification of networks and regions of interest

The two main networks of interest – SN and CEN – were identified using ICA applied to resting-state fMRI data (Figure S2). From the SN ICA map, we identified ROIs in the AI, ACC and VLPFC bilaterally. From the right CEN ICA map, we identified ROIs in the right DLPFC and right PPC. From the left CEN, we identified the left DLPFC and left PPC. The anatomical location of these nodes is shown in Figure S2 and Table S2. Subsequent analyses were based on these five canonical nodes of the SN and right CEN. Our analysis focused primarily on these five right hemisphere ROIs. Additional analyses using ROIs based on regional peaks selected from task-related activation (Figure S3), and findings from homologous left hemisphere ROIs (Figure S4) and sensory ROIs are described in Supplementary Information (Text S1).

### Comparison of task-related activation in children and adults

**Figure 1:**
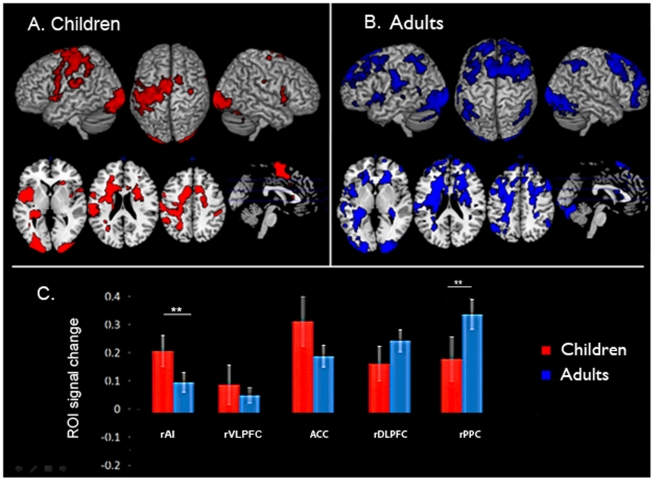
Brain activation in the Salience Network (SN) and Central Executive Network (CEN) during problem solving. (**A**) Children and (**B**) Adults. (**C**) Task-related signal change in ROIs within the SN and CEN. Compared to adults, children showed stronger activation in the right AI and weaker activation in the right PPC (** *p*<0.01, FDR corrected).

We first examined fMRI responses within the five SN and CEN ROIs during the arithmetic problem solving task. Task-related brain activation was identified using a general linear model with arithmetic problem solving task versus rest/null condition contrast. Only correct trials were included in the analysis. All five right-hemisphere nodes showed significant task-related activation in both children and adults (Figure 1A, 1B). Compared to adults, children showed stronger activation in the rAI (*t(38)* = 3.23; *p*<0.01 , FDR corrected) and weaker activation in the rPPC (*t(38)* = 3.41 ; *p*<0.01, FDR corrected) (Figure 1C).

### Comparison of functional connectivity in children and adults

We then examined differences in functional connectivity between children and adults. Functional connectivity here is measured as instantaneous correlations between pairs of ROIs after removal of drift and physiological noise. We found that rAI connectivity with ACC, rDLPFC, and rPPC, and between the rVLPFC and rDLPFC was significantly greater in adults, compared to children (*p*<0.01, FDR corrected). No ROI pairs showed greater functional connectivity in children, compared to adults (*p*<0.01, FDR corrected).

### Latency analysis of regional responses in the SN and CEN

**Figure 2:**
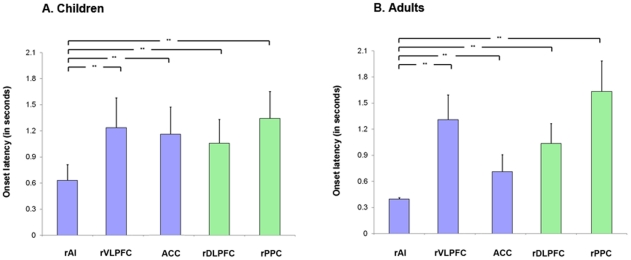
Onset latencies of event-related fMRI responses in the Salience Network (SN) and Central Executive Network (CEN) during problem solving. Onset latencies in the five key nodes of the SN (blue bars), and CEN (green bars) are shown in (**A**) Children and (**B**) Adults. In both groups, the rAI had earlier onset latencies compared to rVLPFC, ACC, rDLPFC, and rPPC (** *p*<0.01, FDR corrected). Compared to adults, rAI onset latencies in children were significantly slower (** *p*<0.01, FDR corrected).

**Figure 3:**
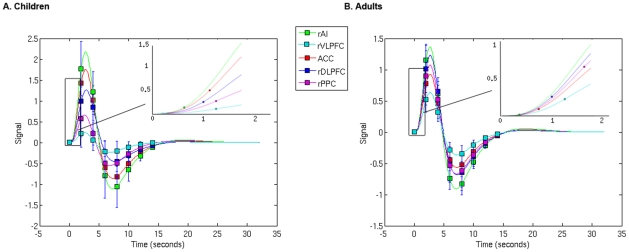
Fitted event-related fMRI responses in the Salience Network (SN) and Central Executive Network (CEN) during problem solving. (**A**) Children and (**B**) Adults. The fMRI BOLD response was fitted using a linear basis function that is a combination SPM canonical hemodynamic response function and a temporal derivative in each of the key nodes of SN and CEN. The fitted responses were averaged across subject for each node plotted against time for each group. Error bars at each TR show standard error of the fitted responses across subjects. Expanded view shows time of onset (shown as filled circle) for each of the key nodes. Onset time was defined as the time at which the slope of the fitted response exceeded 10% of the maximum slope of the ascending part of the response. It can be seen that the rAI had earlier onset latencies compared to rVLPFC, ACC, rDLPFC, and rPPC in children and adults.

We examined differences in the onset latency of the event-related fMRI responses in the five right hemisphere ROIs. We extracted the mean time-course in each ROI, and used a linear basis function that is a combination of the SPM canonical hemodynamic response function and a temporal derivative to fit the event related BOLD response for each subject and event, and then averaged the fitted responses across events and subjects. Onset latencies were then computed as the time point at which the slope of the fitted response reached 10% of its maximum positive (or negative) slope in the initial ascending (or descending) segment [8]. This analysis revealed that the event-related fMRI signal in the rAI has an onset significantly earlier compared to signals in the rVLPFC, ACC, rDLPFC and rPPC (*p*<0.01; FDR corrected), an effect that was observed in both children and adults (Figure 2; Figure 3, Figure S5). The rAI, but not the other four ROIs, had onset latencies significantly earlier in adults, compared to children.

### Causal interaction between nodes of the SN and CEN

We used two different quantitative methods to examine causal interactions in fMRI data. Based on our previous studies, we first used multivariate Granger causal analysis (MGCA) [8] to investigate dynamic interactions between all five right hemisphere ROIs. While there are some concerns that systematic differences across brain regions in hemodynamic lag can potentially lead to spurious estimations of causality [31] recent analyses suggest that when applied at the group level, MGCA has good control over spurious results [32] [33]. Our detailed simulations [34] suggest that MGCA is able to recover causal network structure in spite of the presence of HRF delay confounds. In light of these considerations and other recent discussion about the merits and limitations of MGCA [33], [35] we conducted additional analyses using Multivariate Dynamical Systems (MDS) [34]. MDS is a novel state-space model to estimate intrinsic and experimentally-induced modulatory causal interactions between multiple brain regions that overcomes several limitations of existing methods [34].

**Figure 4:**
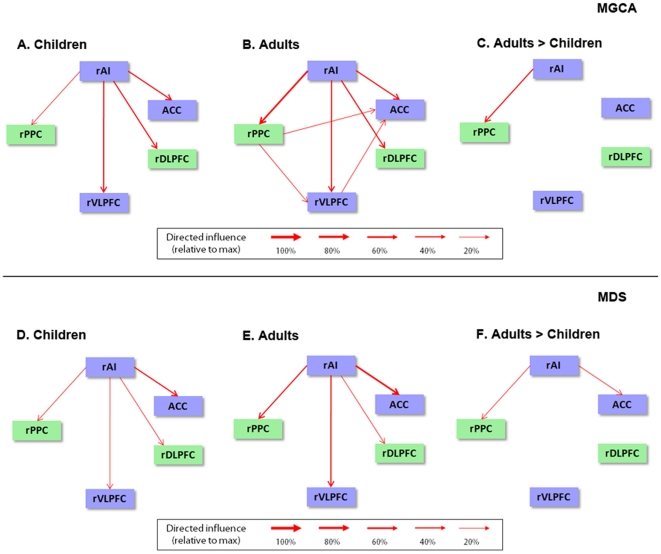
Developmental changes in causal network interactions during problem solving. ***Top row*** **.** Multivariate Granger Causal Analysis (MGCA) of the five key nodes of the Salience network (blue rectangles), and Central Executive network (green rectangles) are shown in (**A**) Children and (**B**) Adults. (**C**) Weaker causal interactions in Children, compared to Adults. ***Bottom row*** **.** Converging evidence from a state-space Multivariate Dynamical Systems (MDS) model [34].

Briefly, MGCA detects causal interactions between brain regions by assessing the relative prediction of signal changes in one brain region based on the time-course of responses in another. We performed MGCA using a multivariate model on the time-courses extracted from each of the ROIs. We used bootstrap techniques to create null distributions of influence terms (F-values) and their differences. In children, MGCA revealed statistically significant direct causal influences from the rAI to the rVLPFC, ACC, rDLPFC, and rPPC (Figure 4A). In adults, MGCA revealed causal influences from the rAI to these same regions (Figure 4B). Quantitative comparison of the strength of causal influences revealed that the strength of interactions from the rAI to rPPC was significantly greater in adults, compared to children (*p*<0.01; FDR corrected), as shown in Figure 4C. No links showed reduced causal influence in adults, compared to children.

An identical set of analyses were conducted using MDS methods which have the advantage of modeling causal interactions in the latent “neuronal” signals, rather than in the fMRI signal itself. Furthermore, MDS also takes into account inter-regional variations in hemodynamic response in an explicit manner [34]. This analysis confirmed results from the MGCA and demonstrate the robustness of our findings (Figure 4D,E,F).

### Graph-theoretical network analysis

**Figure 5:**
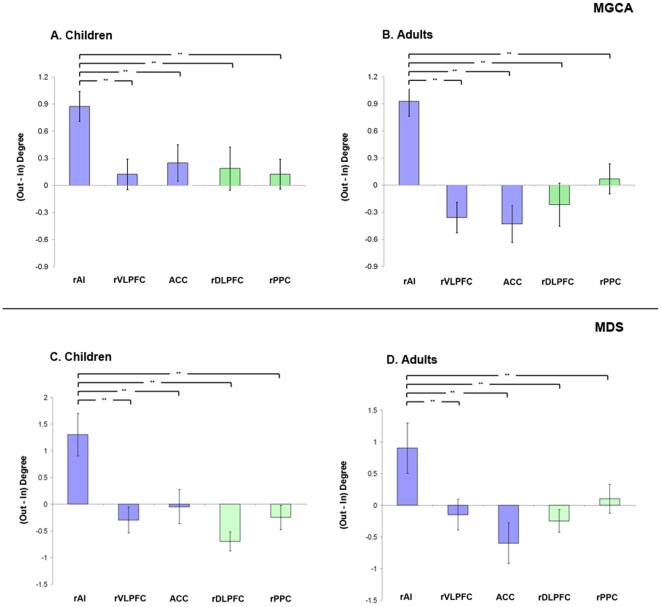
Net outflow of causal network interactions during problem solving in children and adults. ***Top row*** **.** Net causal outflow (out – in degree) in the five key nodes of the Salience Network (blue bars), and Central Executive Network (green bars) derived using Multivariate Granger Causal Analysis (MGCA) are shown (**A**) Children and (**B**) Adults. In both groups, the rAI had significantly higher net causal outflow than the rVLPFC, ACC, rDLPFC, and rPPC (** *p*<0.01, FDR corrected). ***Bottom row*** **.** Converging evidence from a state-space Multivariate Dynamical Systems (MDS) model [34].

To quantify the causal interactions of each node of the network, we performed graph-based network analyses. Analysis of the causal network identified with MGCA revealed that the rAI had the highest number of causal outflow connections (out-degree), the lowest number of causal inflow connections (in-degree), and the shortest path length among all regions. The rAI also had a significantly higher net causal outflow (out-in degree) than all of the other regions (*p*<0.05; FDR corrected). These results were observed in both children and adults, suggesting that the rAI is an outflow hub in both groups (Figure 5A, Figure 5B). There were no group differences in the node-wise net causal outflow nor path length between the groups. Similar graph-based analyses were conducted on causal network identified using MDS. This analysis gave results that were identical to those observed from the MGCA and demonstrate the robustness of our findings (Figure 5C,5D).

### Comparison of rAI-rPPC structural connectivity in children and adults

**Figure 6:**
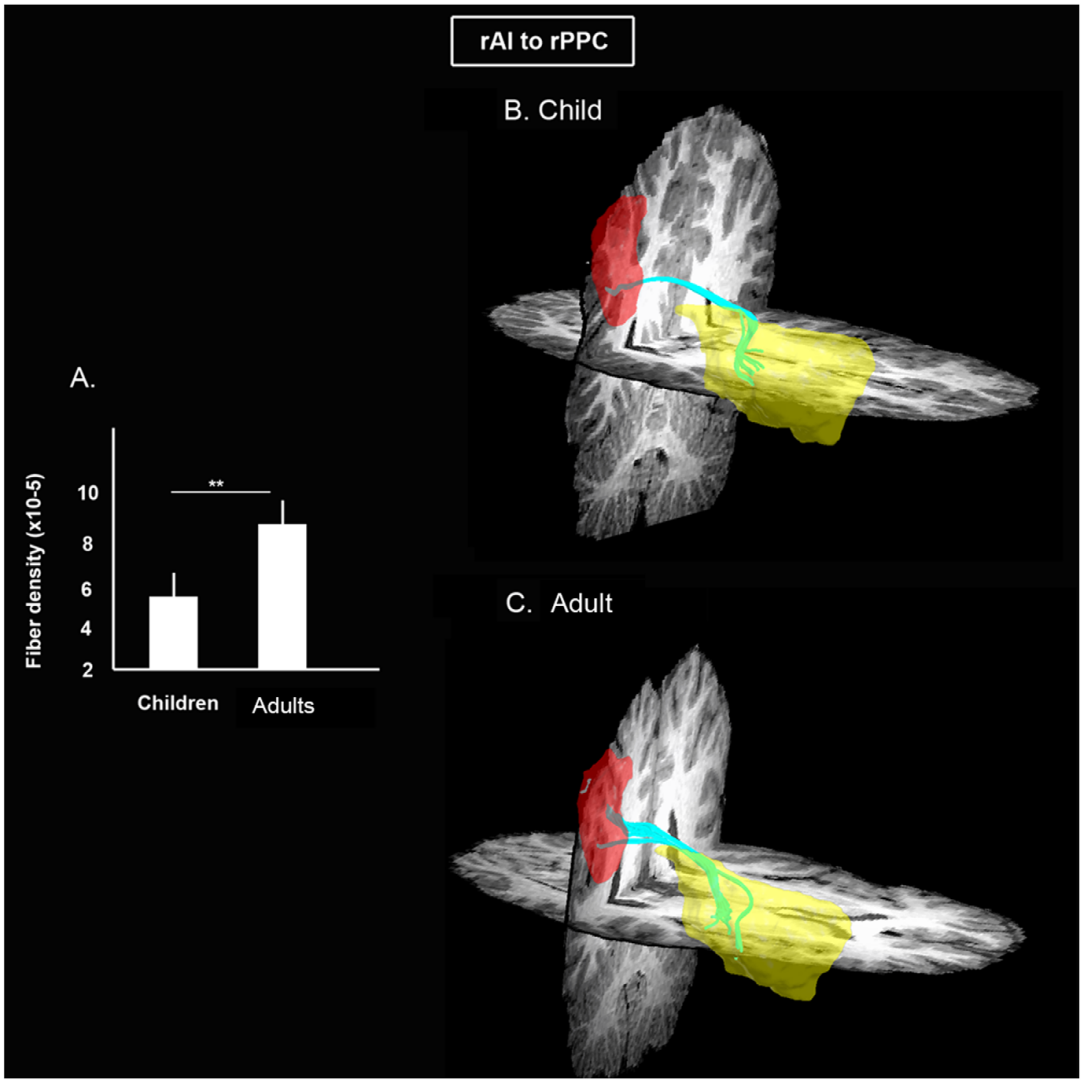
Developmental changes in white matter tracts linking rAI and rPPC. (**A**) Fiber density, the number of fibers per unit area, between the rAI (yellow) to rPPC (red) is significantly lower in children, compared to adults (** *p*<0.01). Tracts connecting rAI to rPPC (cyan tracts) are shown in (**B**) Children and (**C**) Adults.

We used DTI and quantitative tractography to investigate the anatomical correlates of developmental changes in causal interactions between the rAI and rPPC. The density of fibers along the superior longitudinal fasciculus linking the rAI and rPPC was significantly lower in children compared to adults (*p*<0.01), as shown in Figure 6. Mean fractional anisotropy (FA) of rAI-rPPC tracts was also significantly lower in children (*p*<0.01). The rAI-rPPC fibers observed here have been previously identified to be part of the third component of the superior longitudinal fasciculus (SLF III) which connects the rostral part of the inferior parietal lobule with the lateral inferior frontal lobe [36]. Visual inspection of individual subject rAI-PPC SLF III fibers suggest that tracts emanating from magno-cellular supramarginal area (PFm) [37] connect to the mid/posterior aspect of the insula. In the absence of anatomical atlases that clearly demarcate the subregions of insula, a more definitive statement on the exact trajectories of the insula-parietal fibers would require future studies that demarcate insula subregions based on parcellation techniques [38], [39], [40], [41], [42], [43], [44].

### Comparison of structure-function relationships in children and adults

**Figure 7:**
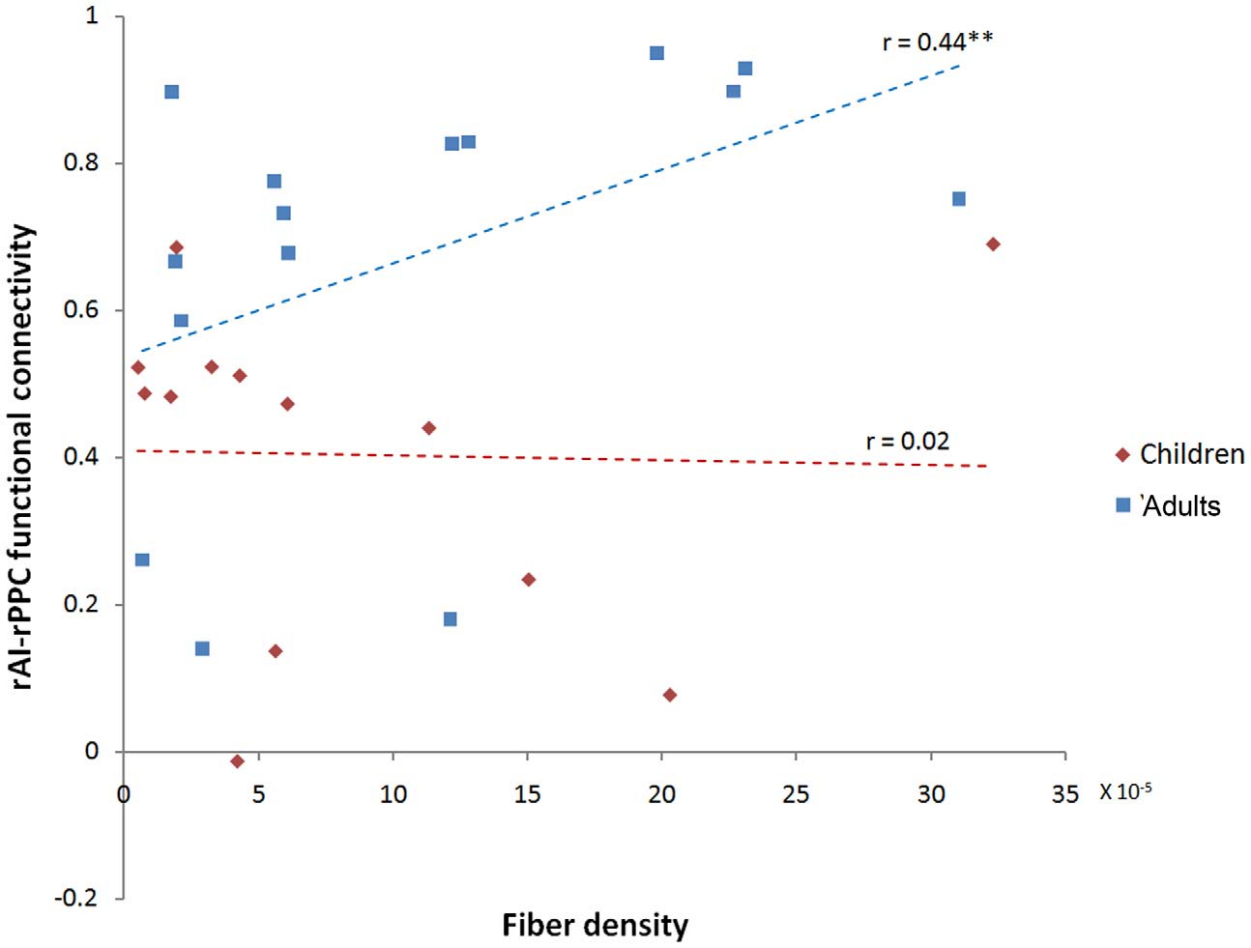
Relation between functional network interactions and underlying white matter tracts in children and adults. Functional interactions is correlated with structural white matter connectivity in Adults (*r* = 0.44; ** *p*<0.05) but not in Children (*r* = 0.02; *p* = 0.9). Functional interactions were assessed using fMRI time-series correlations between the rAI and rPPC. Fiber density was based on DTI tractography.

We compared the relationship between functional and structural connectivity between the rAI to rPPC in children and adults. We found that functional connectivity, measured by instantaneous temporal correlations, and structural connectivity, measured by fiber density, between the rAI to rPPC was significantly correlated in adults (*r* = 0.44; *p*<0.05) but not in children (*r* = 0.02; *p* =  0.9), as shown in Figure 7. Similarly, causal connectivity, measured by causal influence terms were correlated with structural connectivity, measured by fiber density in adults (*r* = 0.23; *p*<0.05) but not in children (*r* = 0.06; *p* = 0.78).

### Relating causal interaction to behavior in children and adults

We used multivariate sparse regression analysis, based on GLMnet [43], to investigate causal network interactions which collectively predict behavior. Causal functional connectivity strength between brain regions was used as predictor variables and either reaction time or accuracy was used as the dependent variable.

#### Reaction time

In children, strength of directed causal influences from rAI to rDLPFC, rAI to ACC, and rAI to rPPC cumulatively predicted reaction time. The remaining seven connections were non-significant (zero) i.e. they did not contribute to the prediction of reaction times. In adults, the strength of directed causal influences from rAI to rPPC and rAI to rVLPFC cumulatively predicted reaction time. The remaining eight connections were non-significant. Comparison of model fit revealed that causal network interactions better predicted reaction time in adults (*R* ^2^ = 0.66, *p*<0.001; mean square error = 0.54), than in children (*R* ^2^ = 0.50, *p*<0.001; mean square error = 0.75) (Table 1).

**Table 1:** Causal network interactions predict behavior differently in children and adults.

	Reaction time				Accuracy			
	Predictive causal connections	R^2^	Mean square error	*p*	Predictive causal connections	R^2^	Mean square error	*p*
*Children*	*rAI→rDLPFC*	0.50	0.75	<0.01	rAI→rACC	0.43	0.83	<0.01
	*rAI→rACC*				rAI→rPPC			
	*rAI→rPPC*				rAI→rVLPFC			
*Adults*	*rAI→rPPC*	0.66	0.54	<0.01	rAI→rACC	0.47	0.76	<0.01
	*rAI→rVLPFC*				rAI→rPPC			

Relationship between cumulative directed causal influences between nodes of the salience and central executive networks and behavior assessed using a multivariate GLMnet model. rAI = right Anterior Insula, ACC = Anterior Cingular Cortex, rVLPFC = right Ventrolateral Prefrontal Cortex, rDLPFC = right Dorsolateral Prefrontal Cortex, rPPC = right Posterior Parietal Cortex. Analysis of GLMnet model fits revealed that causal network interactions better predicted reaction times and accuracy in adults than in children.

**Note:** Connections ordered in decreasing order of importance.

#### Accuracy

In children, strength of directed causal influences from rAI to ACC, rAI to rPPC, and rAI to rVLPFC cumulatively predicted accuracy. The remaining seven connections were non-significant. In adults, the strength of directed causal influences from rAI to rACC, and rAI to rPPC cumulatively predicted reaction time. The remaining eight connections were non-significant. Comparison of model fit revealed that causal network interactions better predicted accuracy in adults (R^2^ = 0.47,p<0.01; mean square error = 0.76) than in children (R^2^ = 0.43, p<0.01; mean square error = 0.83) (Table 1).

Similar results were observed when participant age was included as an additional predictor variable. More specifically, causal network interactions outcompeted age in the prediction of reaction time and accuracy in both children and adults.

## Discussion

We used a novel neurocognitive network approach with multimodal imaging to investigate the maturation of control processes underlying problem solving skills in 7–9 year-old children. Task-independent rsfMRI was first used to identify prominent nodes of the insula-cingulate salience and fronto-parietal central executive networks, two neurocognitive networks implicated in fundamental control processes. Functional connectivity and dynamic causal interactions between the major nodes of these networks were examined during arithmetic problem solving using multiple analytic procedures. The strength of both directed and undirected connections between five key nodes of the SN and CEN was compared between children and adults. Both instantaneous and causal functional connectivity analysis identified the rAI as a locus of immature control signals in children. Furthermore, our study provides novel converging evidence from structural connectivity analysis for weaker white matter pathways underlying fronto-parietal (rAI→rPPC) control signals in children. Remarkably, weaker control signals were associated with lower-levels of task performance in children. Below we describe findings from our quantitative analysis of dynamic functional interactions between key brain areas of insula-cingulate salience and fronto-parietal central executive networks and discuss their implications for understanding the maturation of fundamental control processes in the developing brain [8].

### rAI is a causal outflow hub by age 9

The rAI showed strong causal influences on the ACC node of the SN and the DLPFC and PPC regions of the CEN in children and adults, suggesting that the role of the rAI as a primary node that drives the CEN is established early in development. Two additional analyses were performed to confirm these findings. First, we used a novel state-space MDS model, which estimates causal interactions in latent neuronal signals, rather than the recorded fMRI signals, after taking into account inter-regional variations in hemodynamic response [34]. This analysis confirmed findings based on the MGCA that the rAI has significant causal interactions with several other nodes of the SN and the CEN (Figure 4). Second, a completely different analysis based on the temporal profile of event-related fMRI responses revealed that, in both children and adults, the rAI had the shortest onset latency of all brain regions examined. Graph-theoretic analyses using causal connectivity patterns estimated by MGCA and MDS confirmed that the rAI had the highest net causal outflow and shortest path length of all nodes examined in this study. By definition, nodes which have a higher number of outgoing edges and the shortest path from all other nodes in a graph are referred to as hubs and are thought to play a key role in coordinating information flow [45]. Together, these findings provide converging evidence that the rAI is established as a major causal outflow hub by age 9.

Our findings further suggest a novel pattern of temporal hierarchy among prefrontal and parietal regions implicated in control processes [5], [16], [17]. The rAI emerged as a major source of signals to attentional and working memory systems anchored in the ACC, DLPFC and PPC. Additional follow-up exploratory latency and causal analyses including a sensory ROI along with SEN and CN ROIs suggest that the rAI receives weak input from the sensory cortices, which it further amplifies by exerting top-down control on attentional and working memory systems (see Supplementary Text S1 for details). Critically, our findings indicate that the rAI is the initial locus of control signals, as revealed by converging evidence from causal analysis of ongoing task activity and by onset latency analysis. These results are consistent with previous findings in adults performing auditory and visual attention tasks [8], and extend them to higher-order cognition for the first time in both children and adults. Taken together, these findings suggest that the rAI plays a crucial role as a hub that initiates key control signals during higher-order cognition not only in adults but also in children as young as 9 years of age.

### Immature fronto-parietal causal control signals in children

An important novel finding of our study is that the strength of causal influence from rAI to rPPC was significantly weaker in children, compared to adults. Notably, this group difference was observed using both MGCA and MDS, two different and complementary methods for estimating causal interactions in fMRI data. In addition to differences in the causal interaction between the rAI and rPPC, MDS analysis revealed that the strength of causal influence from rAI to ACC was also significantly weaker in children. Here, we focus on the convergent findings from the two analyses on developmental differences in the causal link from the rAI to the rPPC. The only previous study to have examined developmental changes in causal interactions during cognition did not examine rAI connectivity and no connectivity differences were reported between the extended FIC or any regions of the inferior frontal gyrus with the PPC [46]. In parallel with the causal interactions differences observed in our study, instantaneous functional connectivity between rAI and rPPC regions was also weaker in children. Critically, weak control signals from rAI predicted lower rPPC activation in children. Children showed higher rAI activation and lower rPPC responses than adults, suggesting that the strength of causal interactions from the rAI, rather than overall signal level, is more important for regulating rPPC responses. The PPC node of the CEN examined here was anatomically localized to the supramarginal gyrus, part of the posterior association cortex that helps to maintain task-related representations in working memory during problem solving [47], [48]. In particular, arithmetic problem solving tasks involve dynamic integration of symbolic information within working memory [49], [50], [51], and the right supramarginal gyrus is consistently activated during tasks involving visuo-spatial working memory in both children and adults. Right supramarginal gyrus involvement in working memory is also known to undergo protracted developmental changes from childhood to adulthood [24], [25], [52]. Taken together, these findings suggest weak signaling within the fronto-parietal nodes of the CEN in children negatively impacts the ability to maintain task-relevant representations needed for achieving adult-like levels of performance. Our results extend previous research by showing for the first time that both causal and instantaneous cross-network connectivity is immature in children and that the rAI is a major locus of immature cross-network frontal control signals to the parietal cortex.

### Linking functional and structural connectivity between the rAI and rPPC

Multimodal analysis of fMRI and DTI data revealed that functional connectivity differences between the rAI and rPPC were associated with weak structural links between these areas. Quantitative DTI-based tractography showed that the density of white-matter fiber tracts connecting the rAI and rPPC was significantly lower in children, compared to adults. This result is consistent with previous studies showing slow maturation of long-distance white matter tracts [53], [54], including those linking prefrontal and posterior parietal cortices [55], [56], [57]. We also found that children had lower FA along tracts linking the rAI and rPPC, indicating slow development of microstructural integrity of white matter. Thus, both functional and structural connectivity between rAI and rPPC is significantly weaker in children compared to adults. Notably, we found that both causal and instantaneous measures of functional connectivity between these regions were correlated with structural connectivity in adults. No such relation was observed in children suggesting that function-structure relationships between the rAI and rPPC become more stable with development, consistent with previous evidence of similar patterns of function-structure relationships [58]. Our findings provide the first direct evidence that the development of structural connectivity between the rAI and rPPC may play an important role in the maturation of fronto-parietal control signals.

### Casual network interactions moderate performance

As noted above, children were significantly slower and less accurate than adults. We examined whether this behavioral difference was the result of weak network interactions. We found that the strength of causal network interactions collectively were strongly predictive of reaction times; in contrast, the rAI→rPPC link by itself was only weakly correlated with response latency in children and adults. Using multivariate sparse regression analysis, we found that network interactions better predicted reaction time in both children and adults. In children, the strength of rAI→rPPC along with rAI→rVLPFC collectively predicted reaction times, while in adults the strength of rAI→rPPC along with rAI→ACC and rAI→rDLPFC collectively predicted reaction times. It is noteworthy that even though a different set of links predicted reaction times in both groups, the rAI→rPPC link was common to both. We also found that reaction times were better predicted in adults, compared to children. These results suggest that it is the multiple network interactions as a whole, rather than individual links by themselves, that moderate performance. Critically, similar results were observed when accuracy instead of reaction time was used as the performance measure. Thus, casual interactions between the rAI and rPPC are an important factor for mediating performance improvements in higher-order cognition with development.

### Dissociating primacy of rAI and rVLPFC in control

It is noteworthy that the rAI showed the strongest causal signals, even though the rVLPFC has been most commonly implicated in control [10]. Previous studies, have not however, directly examined causal influences from these two distinct regions of the FIC, and quite often have mislabeled what are clearly AI activations as VLPFC or IFG. To address this issue, in the present study, we separated the FIC into two distinct nodes, one centered in the rAI and the other in the rVLPFC. Our analysis showed that the rAI has an earlier onset and a stronger causal influence on other nodes than the rVLPFC. These results show unequivocally that the AI has strong causal influences on the rVLPFC, reiterating its role as a principal source of prefrontal control signals that precedes the rVLPFC. Furthermore, onset latencies did not significantly differ between rVLPFC and rPPC, although they were significantly different between rAI and rVLPFC and between rAI and rPPC. These results provide further evidence for the primacy of control signals from the rAI, and suggest that systems involved in detecting saliency [12] also play an important role in control. Our findings are consistent with the hypothesis that the rAI plays a more primal role in initiating control signals [18]. We propose that in young children, as in adults, the rAI is critically involved in attentional capture, task-switching and generation of control signals that facilitate access to working memory resources necessary for cognition. From a neurodevelopmental point of view, it is noteworthy that such a control system is already in place by age 9, even though the forward causal paths are not fully mature. Further research is needed to clarify the extent to which these findings hold for other cognitive domains such as response inhibition.

### A neurocognitive network perspective on the development of control

Efficient control requires the concerted coordination between multiple brain regions and there is growing evidence to suggest that this is implemented via dedicated neurocognitive networks [3]. In this study, we used a neurocognitive model for examining the role of key nodes within the insula-cingulate SN and fronto-parietal CEN in fundamental control processes. Importantly, the key nodes of these networks were determined independently using task-free rsfMRI data. The locations of the five major nodes were virtually identical in children and adults and all five right hemisphere frontal and parietal ROIs in the SN and CEN showed significant task-related activation in both groups. The profile of causal interactions observed in our study is particularly noteworthy because the CEN and SN are often co-activated during cognitive tasks in children and adults, and isolating focal responses in a consistent manner from task-based fMRI activations is not straightforward. This is especially true in developmental studies since children tend to show diffuse activations in the prefrontal cortex, making it difficult to disambiguate functional interactions within this cortical region [29]. To circumvent this problem we demarcated specific networks and their nodes using intrinsic connectivity analysis of rsfMRI data [8]. In principle, the nodes of the two networks, and in particular, the AI and PPC could have been chosen in several different ways. Indeed, multiple additional analyses with alternate choices of brain regions demonstrated the same pattern of the results reported here. The network perspective allows us to not only examine developmental changes using a principled approach for characterizing brain systems but also has the advantage of integrating the present study with an emerging literature on insula-cingulate and fronto-parietal circuits involved in fundamental aspects of control in the human brain [4], [8], [18].

A unified network approach – wherein we first specify intrinsic brain networks using rsfMRI data and then analyze interactions among anatomically discrete regions within these networks during cognitive information processing – enhances our ability to generalize beyond individual task activated foci and also provides a common framework for comparing brain response and connectivity in children and adults. Our findings are likely to have important implications for understanding the development of control mechanisms subserved by dynamic interactions between neurocognitive networks. Further studies are needed to examine whether similar control mechanisms underlie the functional maturation of specific cognitive processes involving inhibition, memory and decision-making. The quantitative approach developed here is likely to be useful in the investigation of neurodevelopmental disorders, such as autism and attention deficit/hyperactivity disorder, in which control processes are impaired.

## Materials and Methods

### Participants

Twenty-three children and twenty-two IQ-matched adults participated in this study after providing written informed consent. For those subjects who were unable to give informed consent, written, informed consent was obtained from their legal guardian. The study protocol was approved by the Stanford University Institutional Review Board. Children (10 males, 13 females) ranged in age from 7 to 9 (mean age 7.95) with an IQ range of 88 to 137 (mean IQ: 112). Adults (11 males, 11 females) ranged in age from 19 to 22 (mean age 20.4) with an IQ range of 97 to 137 (mean IQ: 112).

### Functional MRI

The fMRI experiment consisted of 52 arithmetic problems presented in a jittered event-related design along with “rest” or “null” trials in which participants passively viewed a cross on the screen. In the arithmetic trials, participants were presented with an equation involving two addends and a resultant and were asked to indicate via a button box whether the resultant was correct or incorrect. Half the addition trials consisted of problems with addends different from ‘1’ (e.g. 3+4 = 7). One operand ranged from 2 to 9, the other from 2 to 5 (tie problems such as ‘5+5 = 10’, were excluded), and resultants were correct in 50% of the trials. Incorrect answers deviated by ±1 or ±2 from the correct sum. The other half of the addition trials had the same format but one addend was ‘1’ (e.g. 5+1 = 7). Stimuli were displayed for 5 seconds with an inter-trial interval of 500 msec followed by a blank screen for 500 msec and an inter-trial jitter that varied between 0 to 3500 msec with an average duration of 1814 msec. Each subject underwent a math task scan and 8-min resting-state scan.

fMRI data acquisition, preprocessing, analysis of task data with General Linear Model (GLM), Independent component analysis (ICA) of resting data, and functional connectivity analysis procedure is described in detail in Supplementary Information (Text S1). Here we describe methods specifically related to analysis of causal interactions.

#### Regions of Interest selection

We defined regions of interest (ROIs) in five key nodes of the SN, right CEN, and left CEN based on the peaks of the ICA clusters. ROIs were selected from respective combined-group ICA clusters: in the rAI, VLPFC and ACC (on the SN ICA map); in the rDLPFC and rPPC (on the right CEN ICA map); in the lDLPFC and lPPC (on the left CEN ICA map). After visually selecting a voxel with the highest *Z* score within each cluster on the functional map, the ROIs were constructed by drawing spheres with centers as the seed-point and a radius of 8 mm.

#### Multivariate Granger causal analysis

MGCA was performed in accordance with the methods of Seth et al. [59]. First, the mean time course from each ROI was extracted for all subjects. Each time series was then detrended and its temporal mean was removed. MGCA was performed to test for causal influences between ROIs using difference of influence (doi) terms (F_x→y_ – F_y→x_). We performed statistical inferencing on the causal connections using non-parametric analysis. Empirical null distribution of influence terms and their differences were estimated by generating surrogate datasets under the null hypothesis that there are no causal interactions between the regions. Those directed connections whose mean (across subjects in the group) was significantly different from the mean of the null distribution were identified using statistical tests and a stringent threshold (*p*<0.01, FDR corrected). The stringent threshold was chosen to avoid potentially spurious causal links introduced by the low temporal resolution and hemodynamic blurring in the fMRI signal. Between group differences in the causal connectivity graphs were determined as links whose mean difference in the doi term significantly differed from mean of the null distribution of difference of doi terms (*p*<0.01, FDR corrected).

#### Multivariate Dynamical Systems (MDS) analysis

Multivariate Dynamical Systems (MDS) is a novel state-space Multivariate Dynamical Systems (MDS) model to estimate intrinsic and experimentally-induced modulatory causal interactions between multiple brain regions [34]. It overcomes several limitations of existing methods. First, the mean time course from each ROI was first extracted for all subjects. Each time series was then detrended and its temporal mean was removed. From the regional fMRI timeseries data, the MDS model estimates the contextually induced causal interactions between brain regions while accounting for variations in hemodynamic responses in these regions. Notably, the MDS model can be seen as an extension of the multivariate granger causal analysis (GCA) wherein a vector autoregressive model for latent, rather than BOLD-fMRI, signals are used to model the causal interactions among brain regions. Furthermore, the MDS model also takes into account variations in the HRF as well as the influences of modulatory and external stimuli in estimating causal interactions between brain regions. In brief, the MDS approach models the multivariate fMRI time series by the following state-space equations:
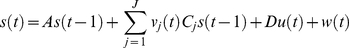
(1)

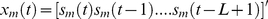
(2)

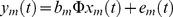
(3)In Equation (1), 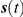 is a 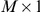 vector of latent signals at time *t* of M regions, 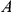 is an 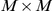 connection matrix wherein 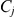 is an 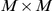 connection matrix ensued by modulatory input 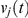, *J* is the number of modulatory inputs. The non-diagonal elements of 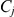 represent the coupling of brain regions in the presence of modulatory input 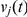. 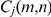 denotes the strength of causal connection from n-th region to m-th region. Therefore, latent signals ***s*** *(t)* in *M* regions at time *t* is a bilinear function of modulatory inputs 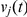 and its previous state ***s*** *(t−1)*. 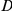 is an 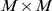 diagonal matrix wherein 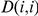 denotes external stimuli strength to i-th region. 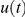 is an 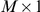 binary vector whose elements represent the external stimuli to m-th region under investigation. 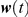 is an 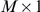 state noise vector whose distribution is assumed to be Gaussian distributed with covariance matrix *Q*(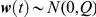). Additionally, state noise vector at time instances *1,2,….,T* (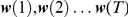) are assumed to be identical and independently distributed (iid). Equation (1) represents the time evolution of latent signals in M brain regions. More specifically, the latent signals at time *t*, 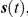 is expressed as a linear combination of latent signals at time *t−1*, external stimulus at time t (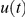), bilinear combination of modulatory inputs 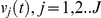 and its previous state, and state noise 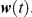 The latent dynamics modeled in Equation (1) gives rise to observed fMRI time series represented by Equations (2) and (3).

We model the fMRI time series in region *“m”* as a linear convolution of HRF and latent signal 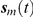 in that region. To represent this linear convolution model as an inner product of two vectors, the past *L* values of 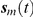 are stored as a vector. 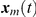 in equation (2) represents an 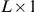 vector with L past values of latent signal at m-th region.

In Equation (3), 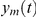 is the observed BOLD signal at *t* of *m*-th region. 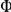 is a 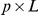 matrix whose rows contain bases for HRF. 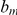 is a 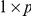 coefficient vector representing the weights for each basis function in explaining the observed BOLD signal 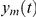. Therefore, the HRF in *m-th* region is represented by the product 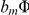. The BOLD response in this region is obtained by convolving HRF 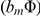 with the *L* past values of the region's latent signal 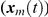 and is represented mathematically by the vector inner product 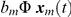. Uncorrelated observation noise 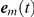 with zero mean and variance 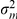 is then added to generate the observed signal 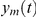. 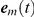 is also assumed to be uncorrelated with 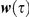, at all *t* and 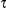 .Equation (3) represents the linear convolution between the embedded latent signal 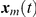 and the basis vectors for HRF. Here, we use the canonical HRF and its time derivative as bases, as is common in most fMRI studies. Equations (1–3) together represent a state-space model for estimating the causal interactions in latent signals based on observed multivariate fMRI time series.

Estimating causal interactions between *M* regions specified in the model is equivalent to estimating the parameters 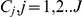. In order to estimate 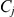
's, the other unknown parameters 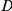, *Q*, 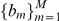 and 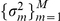 and the latent signal 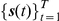 based on the observations 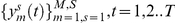, where *T* is the total number of time samples and *S* is number of subjects, needs to be estimated. We use a variational bayes approach (VB) for estimating the posterior probabilities of the unknown parameters of the MDS model given fMRI time series observations for *S* number of subjects.

The statistical significance of the causal connections was assessed by using non-parametric analysis. Empirical null distribution of the parameters 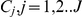 was estimated by generating surrogate datasets under the null hypothesis that there are no causal interactions between the regions. Those directed connections whose mean (across subjects in the group) was significantly different from the mean of the null distribution were identified using statistical tests and a stringent threshold (*p*<0.01, FDR corrected). Between group differences in the causal connectivity graphs were determined as links whose mean difference in the 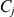 significantly differed from mean of the null distribution of difference of 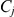 (*p*<0.01, FDR corrected).

#### Graph-theoretical network analysis

To describe the interactions between brain regions in the causal network generated by MGCA, we examined the following graph metrics: (1) Out-degree: Number of causal outflow connections from a node in the network to any other node. (2) In-degree: Number of causal in-flow connections to a node in the network from any other node. (3) (Out – In) degree: Difference between out-degree and in-degree is a measure of the net causal outflow from a node. (4) Path length: Shortest path from a node to every other node in the network (normalized by the number of nodes minus one). Shorter path lengths indicate a more strongly interconnected or “hub-like” node. A two-sample *t*-test was then applied on two key network metrics, the (out-in) degree and the path length to identify those nodes whose network metrics were significantly different from the other nodes.

#### Regression analysis

To investigate whether causal network interactions predict behavior differently in children and adults, we examined causal connectivity patterns in the two groups. The causal functional connectivity patterns – strength of causal connectivity of 10 pairs of anatomical regions – along with behavioral measures were used as the input to a sparse regression algorithm. The sparse regression algorithm identifies causal network connections that predict behavior by modeling the relationship between the dependent variable (RT) and the independent variables (strength of pair-wise causal connectivity). An added advantage of using a sparse regression algorithm, as opposed to traditional regression, is that it performs feature selection wherein the coefficients of independent variables that do not contribute to the prediction of the dependent variable are set to zero. In our case, this entails that the regression analysis would identify the causal network connections that predict behavior while the non-contributing connections would be set to zero. Such sparse methods are particularly elegant when the number of possible predictor variables is large. In sum, we used sparse regression analysis instead of the more conventional regression analysis so that we could not only investigate whether causal connectivity predicts behavioral measure(s) but also identify in a purely data-driven manner which subset of causal connections, if any, predicts behavior. GLMNet [43], a widely used sparse-regression algorithm was used in our analysis. Mean square error was used to measure the performance of the regression algorithm in predicting behavior. The sparse regression analysis was performed separately for each group.

### Diffusion Tensor Imaging

DTI data was obtained from 18 of the 23 children subjects and 15 of 22 adults. Acquired images underwent the following preprocessing steps: eddy-current correction, alignment with T1-weighted anatomical images, resampling, and tensor computation. Fiber tracts between the rAI and rPPC were computed as previously described [58]. For each subject, the density and mean fractional anisotropy of the fibers connecting the rAI to the rPPC was measured, in native space (see Supplementary Text S1 for details). In this study, we used fiber density and mean FA as measures of the integrity of the fiber tracts of interest.

## Supporting Information

Figure S1 **Accuracy and reaction time during problem solving.** (**A**) Accuracy was significantly lower in children, compared to adults (** *p*<0.01). (**B**) Reaction times were significantly higher in children, compared to adults (** *p*<0.01). Mean and standard error are shown.
(TIF)

Figure S2 **Major nodes of the Salience Network (SN) and Central Executive Network (CEN).** SN and CEN networks were derived from combined group ICA of resting-state fMRI data. 8 mm spheres depicting (**A**) Key nodes of the SN include the right anterior insula (rAI), right ventrolateral prefrontal cortex (rVLPFC), and anterior cingulate cortex (ACC). (**B**) Key nodes of the CEN include the right dorsolateral prefrontal cortex (rDLPFC) and right posterior parietal cortex (rPPC).
(TIF)

Figure S3 **Developmental changes in network interactions during problem solving.** In this case, ROIs were derived from peak task-related activation. Multivariate Granger Causal analysis (MGCA) of the five key nodes of the Salience Network (blue rectangles), and Central Executive Network (green rectangles). ROIs were derived from peak task-related activation (**A**) Children, (**B**) Adults and (**C**) Weaker causal interactions in Children, compared to Adults.
(TIF)

Figure S4 **Developmental changes in network interactions during problem solving.** In this case, left hemisphere ROIs were used. Multivariate Granger Causal analysis (MGCA) of the five key left hemisphere nodes of the Salience Network (blue rectangles), and Central Executive Network (green rectangles) are shown in (**A**) Children and (**B**) Adults. (**C**) No differences were observed in any of these left hemisphere regions.
(TIF)

Figure S5 **Mean raw event-related fMRI signal timeseries in the Salience Network (SN) and Central Executive Network (CEN) during problem solving.** (**A**) Children and (**B**) Adults. Error bars show standard error of the raw event-related fMRI signal timeseries across trials and subjects.
(TIF)

Table S1 **Participant characteristics.** Children and adults did not differ on IQ or gender, while age and years of education were significantly different (** *p*<0.01).
(DOC)

Table S2 **Regions of interest.** ROIs were chosen based on nodes identified in the Salience Network (SN) and the right Central Executive Network (CEN). SN and CEN networks were derived from combined group ICA of resting state fMRI data.
(DOC)

Text S1 **Experimental procedures.**
(DOC)
