# Supplementary figures and images for: Developmental Maturation of Dynamic Causal Control Signals in Higher-Order Cognition: A Neurocognitive Network Model
Source: PLoS Comput Biol. 2012 Feb 2;8(2):e1002374. doi: 10.1371/journal.pcbi.1002374 (PMC3271018; doi:10.1371/journal.pcbi.1002374)

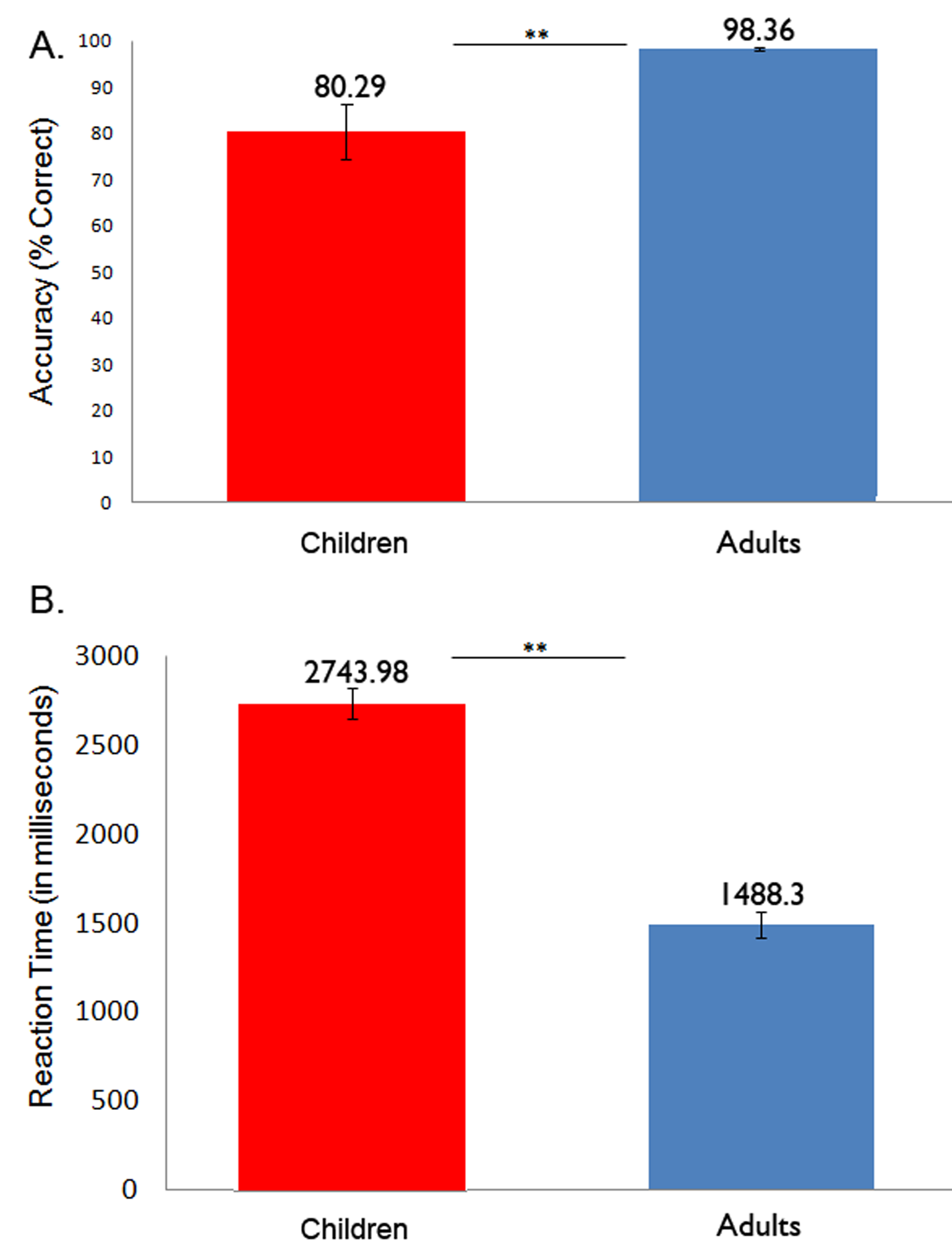

Supplement: Figure S1 — Accuracy and reaction time during problem solving. (A) Accuracy was significantly lower in children, compared to adults (** p<0.01). (B) Reaction times were significantly higher in children, compared to adults (** p<0.01). Mean and standard error are shown. (TIF) [file pcbi.1002374.s001.tif]

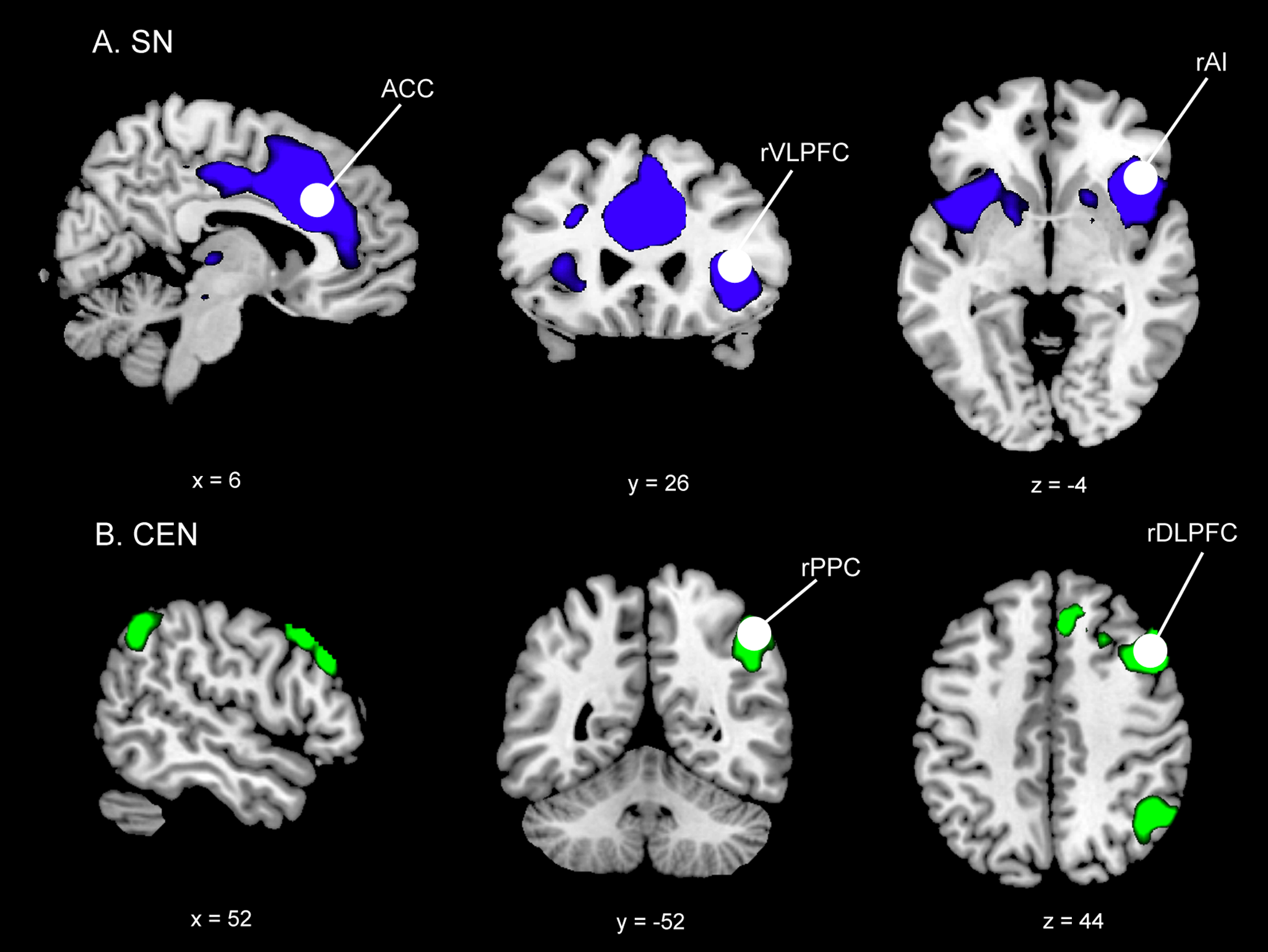

Supplement: Figure S2 — Major nodes of the Salience Network (SN) and Central Executive Network (CEN). SN and CEN networks were derived from combined group ICA of resting-state fMRI data. 8 mm spheres depicting (A) Key nodes of the SN include the right anterior insula (rAI), right ventrolateral prefrontal cortex (rVLPFC), and anterior cingulate cortex (ACC). (B) Key nodes of the CEN include the right dorsolateral prefrontal cortex (rDLPFC) and right posterior parietal cortex (rPPC). (TIF) [file pcbi.1002374.s002.tif]

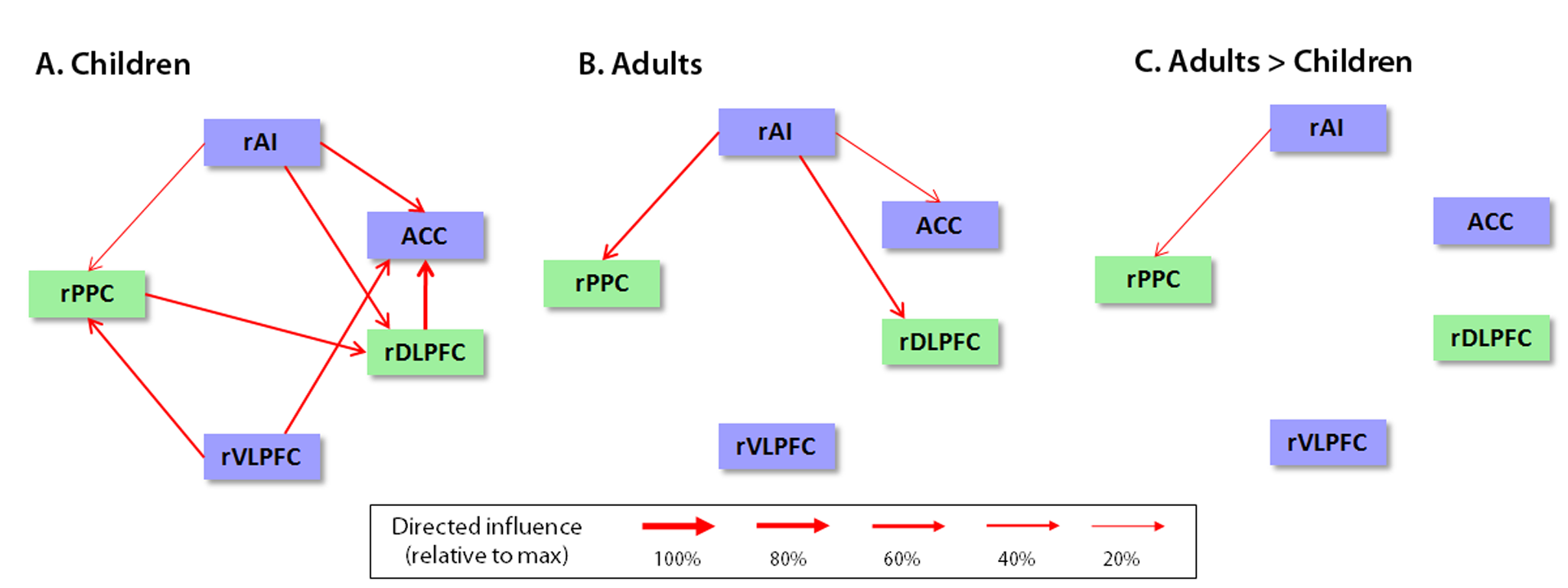

Supplement: Figure S3 — Developmental changes in network interactions during problem solving. In this case, ROIs were derived from peak task-related activation. Multivariate Granger Causal analysis (MGCA) of the five key nodes of the Salience Network (blue rectangles), and Central Executive Network (green rectangles). ROIs were derived from peak task-related activation (A) Children, (B) Adults and (C) Weaker causal interactions in Children, compared to Adults. (TIF) [file pcbi.1002374.s003.tif]

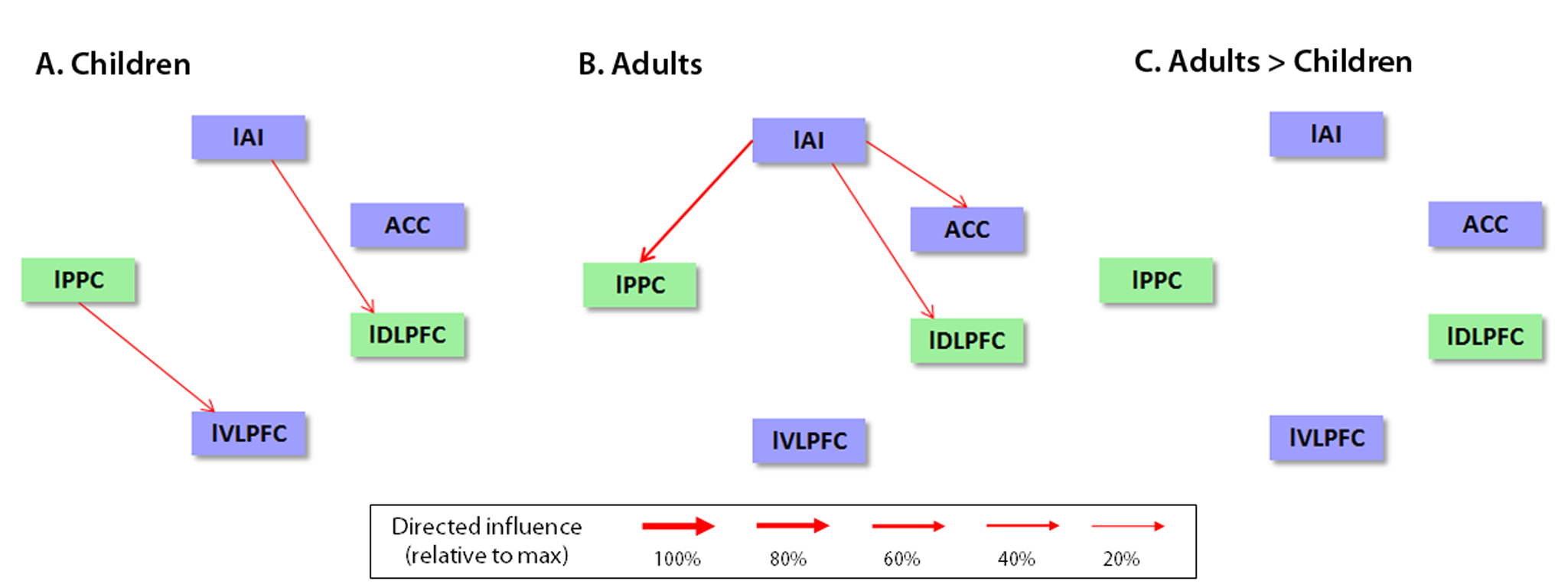

Supplement: Figure S4 — Developmental changes in network interactions during problem solving. In this case, left hemisphere ROIs were used. Multivariate Granger Causal analysis (MGCA) of the five key left hemisphere nodes of the Salience Network (blue rectangles), and Central Executive Network (green rectangles) are shown in (A) Children and (B) Adults. (C) No differences were observed in any of these left hemisphere regions. (TIF) [file pcbi.1002374.s004.tif]

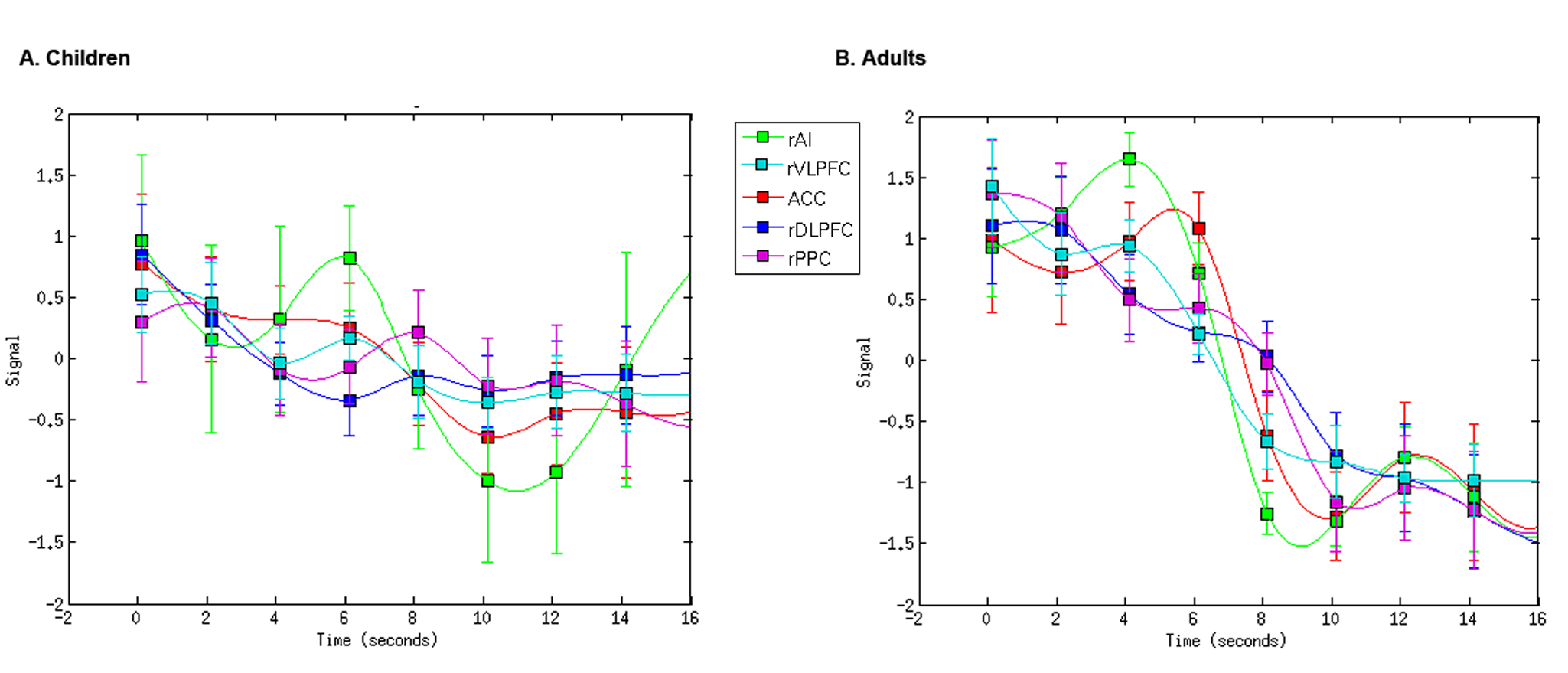

Supplement: Figure S5 — Mean raw event-related fMRI signal timeseries in the Salience Network (SN) and Central Executive Network (CEN) during problem solving. (A) Children and (B) Adults. Error bars show standard error of the raw event-related fMRI signal timeseries across trials and subjects. (TIF) [file pcbi.1002374.s005.tif]
