# Supplementary material for: Developmental Maturation of Dynamic Causal Control Signals in Higher-Order Cognition: A Neurocognitive Network Model
Source: PLoS Comput Biol. 2012 Feb 2;8(2):e1002374. doi: 10.1371/journal.pcbi.1002374 (PMC3271018; doi:10.1371/journal.pcbi.1002374)
Supplement: Table S1 — Participant characteristics. Children and adults did not differ on IQ or gender, while age and years of education were significantly different (** p<0.01). (DOC) [file pcbi.1002374.s006.doc]

**Table S1. Participant characteristics.** Children and adults did not differ on IQ or gender, while age and years of education were significantly different (** *p* < 0.01).

|  | **Children (n = 23)** | **Adults (n = 22)** |
| --- | --- | --- |
| Age | 7.95** (range: 7 to 9) | 20.40** (range: 19 to 22) |
| Gender | 10 males, 13 females | 11 males, 11 females |
| IQ | 112 (range: 88 to 137) | 112 (range:97 to 137) |
| Years of education | 2.52** (range: 2 to 3) | 14.5** (range: 13 to 16) |
