# Supplementary material for: Developmental Maturation of Dynamic Causal Control Signals in Higher-Order Cognition: A Neurocognitive Network Model
Source: PLoS Comput Biol. 2012 Feb 2;8(2):e1002374. doi: 10.1371/journal.pcbi.1002374 (PMC3271018; doi:10.1371/journal.pcbi.1002374)
Supplement: Table S2 — Regions of interest. ROIs were chosen based on nodes identified in the Salience Network (SN) and the right Central Executive Network (CEN). SN and CEN networks were derived from combined group ICA of resting state fMRI data. (DOC) [file pcbi.1002374.s007.doc]

**Table S2. Regions of interest.** ROIs were chosen based on nodes identified in the Salience Network (SN) and the right Central Executive Network (CEN). SN and CEN networks were derived from combined group ICA of resting state fMRI data.

| **Regions of interest** | **Network** | **BA** | **MNI**  **coordinates (mm)** | **Z-score** |
| --- | --- | --- | --- | --- |
| R Anterior Insula (AI) | SN | 47 | 39 23 -4 | 11.66 |
| R Ventrolateral Prefrontal Cortex (VLPFC) | SN | 45 | 42 26 14 | 10.78 |
| R/L Anterior Cingulate Cortex (ACC) | SN | 24/30 | 6 24 32 | 13.16 |
| R Dorsolateral Prefrontal Cortex (DLPFC) | CEN | 9 | 46 20 44 | 14.47 |
| R Posterior Parietal Cortex (PPC) | CEN | 40 | 52 -52 50 | 11.06 |
