# Supplementary material for: Developmental Maturation of Dynamic Causal Control Signals in Higher-Order Cognition: A Neurocognitive Network Model
Source: PLoS Comput Biol. 2012 Feb 2;8(2):e1002374. doi: 10.1371/journal.pcbi.1002374 (PMC3271018; doi:10.1371/journal.pcbi.1002374)
Supplement: Text S1 — Experimental procedures. (DOC) [file pcbi.1002374.s008.doc]

**Supplementary Information**

**Methods**

**Functional MRI**

Data acquisition. Functional Images were acquired on a 3T GE Signa scanner (General Electric, Milwaukee, WI) using a custom-built head coil. Head movement was minimized during scanning by a comfortable custom-built restraint. A total of 29 axial slices (4.0mm thickness, 0.5mm skip) parallel to the anterior commissure(AC)-posterior commissure(PC) line and covering the whole brain were imaged with a temporal resolution of 2 seconds using a T2* weighted gradient echo spiral in-out pulse sequence with the following parameters: TR = 2000msec, TE = 30msec, flip angle = 80°, 1 interleave. The field of view was 20 cm, and the matrix size was 64x64, providing an in-plane spatial resolution of 3.125 mm.  To reduce blurring and signal loss arising from field inhomogeneities, an automated high-order shimming method based on spiral acquisitions was used before acquiring functional MRI scans.

Data preprocessing. A linear shim correction was applied separately for each slice during reconstruction using a magnetic field map acquired automatically by the pulse sequence at the beginning of the scan . Functional MRI data were then analyzed using SPM5 analysis software (http://www.fil.ion.ucl.ac.uk/spm). Images were realigned to correct for motion, corrected for errors in slice-timing, spatially transformed to standard stereotaxic space (based on the Montreal Neurologic Institute (MNI) coordinate system), resampled every 2 mm using sinc interpolation and smoothed with a 6mm full-width half-maximum Gaussian kernel to decrease spatial noise prior to statistical analysis. Translational movement in millimeters (x, y, z) and rotational motion in degrees (pitch, roll, yaw) was calculated based on the SPM5 parameters for motion correction of the functional images in each subject. No participants had a range of movement greater than 3mm translation or 3 degrees of rotation. Motion parameters did not differ between children and adults.

GLM analysis. Task-related brain activation was identified using a general linear model. For the math task, brain activity related to the task condition was modeled using boxcar functions with the SPM canonical hemodynamic response function and a temporal derivative to account for voxelwise latency differences in hemodynamic response. Low frequency drifts at each voxel were removed using a highpass filter (0.5 cycles/min), and serial correlations were accounted for by modeling the fMRI time series as a first degree autoregressive process. A voxelwise *t-*statistics map for the task condition was generated for each participant using an ANOVA on the respective contrast images.

A group-level analysis for the task condition was performed by entering the individual-subject contrast maps into a random-effects analysis, for each group. The resulting voxelwise t statistic maps were used to determine group-level activation for each comparison. Significant clusters of activation were determined using a voxelwise statistical height threshold of (*p* < 0.01), with FWE corrections for multiple spatial comparisons (*p* < 0.01). In addition, a similar analysis was performed to compute between group level differences. Activation foci were superimposed on high-resolution T1-weighted images, and their locations were interpreted using known neuroanatomical landmarks. MNI coordinates were transformed to Talairach coordinates using a nonlinear transformation. In our task-based fMRI analyses we only examined correct trials primarily to focus on neural processes involved in arithmetic problem solving, to rule out the effect of variable neural processing involved in wrong arithmetic responses, and more importantly to match the children group with adults who had very few wrong responses.

ICA analysis. Each participant’s smoothed, normalized images were concatenated across time to form a four dimensional matrix using FSL 3.3. This four dimensional matrix was then analyzed with FSL 4.4 melodic ICA concatenated across participants. This analysis was limited to output only 25 components for the group. From these components, networks of interest – the salience network (SN), right executive control network (right CEN), and left executive control network (left CEN) – were selected for subsequent analyses using previously validated methods . These components were then binarized using SPM5 in order to create templates for choosing network components for individuals.

Each participant’s smoothed, normalized, four dimensional matrix was analyzed with FSL melodic ICA version 12. The number of components output by ICA was determined automatically by the PCA process of the melodic software. For adults, the number of ICA components generated ranged from 23 to 93. For children, the number of ICA components generated ranged from 45 to 74. Children and adults did not differ in the number of ICA components generated. The templates created above for the three networks were then applied to each participant’s individual ICA components to select the “best-fit” network component. To do this, we developed a nonlinear template-matching procedure that involved taking the average z-score of voxels falling within the template minus the average z-score of voxels outside the template and selecting the component in which this difference (the goodness-of-fit) was the greatest. Z-scores here reflect the degree to which a given voxel’s time series correlates with the time series corresponding to the specific ICA component. This method allowed us to select each individual’s SN, right CEN, and left CEN.

Combined-group analyses were performed using the individual best-fit network components for the three networks. Individual *t*-statistics images from both groups were used to determine combined group-level statistical maps using a one sample t-test as implemented in SPM5. The results were masked with a skull-stripped binary image of the standard MNI T1. Significant clusters were determined using a voxel-wise statistical height (*p* < 0.001) and extent (*p* < 0.001) thresholds, corrected at the whole-brain level.

Onset latency analysis. This method provides an independent assessment of relative timing of event-related fMRI responses to task stimuli in each ROI. Critically, latency analysis has been used in previous studies to quantify differences in the onset of the underlying neural activity [4,5,6]. We calculated onset latency by fitting the BOLD response as a linear basis function that is a combination SPM canonical hemodynamic response function and a temporal derivative [7]. The mean timeseries extracted from each ROI for each subject was fitted with this linear function. Onset latencies were defined as the time at which the slope of the fitted response exceeded 10% of the maximum slope of the ascending part of the response [8]. We then performed a two-sample t-test to identify brain regions significantly differing in the onsets of their neural activity (*p* < 0.05, FDR corrected for multiple comparison), for the two groups. In addition, we performed a between group two sample *t*-test to identify pairs of brain regions that showed significant group differences in their onset differences (*p* < 0.05, FDR corrected for multiple comparison).

**Structural MRI**

Data acquisition. For each subject, a high resolution T1-weighted spoiled grass gradient recalled (SPGR) inversion recovery 3D MRI sequence was acquired to facilitate anatomical localization of functional data and for performing anatomical morphometric analyses. The following parameters were used: TI = 300 msec, TR = 8.4 msec; TE = 1.8 msec; flip angle = 15^o^; 22 cm field of view; 132 slices in coronal plane; 256 x 192 matrix; 2 NEX, acquired resolution = 1.5 x 0.9 x 1.1 mm. Structural and functional images were acquired in the same scan session.

**Diffusion Tensor Imaging**

Data acquisition. DTI data was obtained from 18 of the 23 children subjects and 15 of 22 adults. The DTI pulse sequence was a diffusion-weighted single-shot spin-echo, echo planar imaging sequence (TE = 70.8 ms; TR = 8.6 s; field of view = 220 mm; matrix size = 128 x 128; bandwidth = ±110kHz; partial k-space acquisition). We acquired 63 axial, 2-mm thick slices (no skip) for 2 *b* values, b = 0 and b = approximately 850 s/mm^2^. The high *b* value was obtained by applying gradients along 23 different diffusion directions. Two gradient axes were energized simultaneously to minimize TE. The polarity of the effective diffusion-weighting gradients was reversed for odd repetitions to reduce cross-terms between diffusion gradients and imaging and background gradients. Although Jones [9] suggests that measuring more diffusion directions would be a more efficient way to reliably estimate diffusion tensors of arbitrary orientation, our signal-to-noise ratio is sufficiently high from our 4 repeats to produce very reliable tensor estimates suitable for tractography.

Data preprocessing DTI data were preprocessed using a custom program based on normalized mutual information that removed eddy current distortion effects and determined a constrained nonrigid image registration of all the diffusion images [10]. The 6 elements of the diffusion tensor were determined by multivariate regression [11,12]. For each subject, the non–diffusion-weighted (*b* = 0) images were coregistered to the T1-weighted 3-D SPGR anatomical images using a mutual information 3-D rigid-body coregistration algorithm from SPM5. Several anatomical landmarks, including the anterior commissure (AC), the posterior commissure (PC), and the midsagittal plane, were identified by hand in the T1 images. With these landmarks, we computed a rigid-body transform from the native image space to the conventional AC-PC–aligned space. The DTI data were then resampled to this AC-PC–aligned space with 2-mm isotropic voxels using a spline-based tensor interpolation algorithm [13], taking care to rotate the tensors to preserve their orientation with respect to the anatomy [14]. The T1 images were resampled to AC-PC–aligned space with 1-mm isotropic voxels. We confirmed by visual inspection of each dataset that this coregistration technique aligns the DTI and T1 images to within 1–2 millimeters in the brain regions of interest.

Tractography Using custom DTI analysis software (available for download at <http://sirl.stanford.edu/software/>), the tractography procedure was initiated by whole-brain fiber tracking in native space that produced many fiber paths. For tractography purposes, larger anatomical atlas based ROIs that encompassed the smaller 8mm functional ROIs were used. The right insula ROI defined by AAL atlas [15] and the right magno- cellular supramarginal area (PFm) defined by Caspers and colleagues [16] were selected to represent rAI and rPPC ROIs respectively. The right insula ROI defined by AAL atlas, which includes the anterior and posterior areas of the insula, was selected to represent rAI. We chose AAL-defined ROI instead of a Freesurfer segmented ROI because Freesurfer is known to significantly underestimate Insula and for deterministic tractography purposes it is desirable to have larger ROIs as fiber tracking becomes unreliable in gray matter. Additionally, in our previous work, we have successfully identified numerous fiber tracts including anterior angular gyrus to hippocampus, posterior angular gyrus to caudate, and intraparietal sulcus to insula using AAL-defined ROIs [17]. The larger ROIs in MNI space were then warped back to each individual brain to be used for the subsequent DTI tractography analyses. This was done by applying the inverse of the spatial normalization transformation. As fiber tracking becomes unreliable in gray matter, we ensured that our ROIs extended 2–3mm into the white matter. Tracts that did not end in or pass through both ROIs were discarded. Each fiber tract was estimated using a deterministic streamline tracking algorithm [18,19,20] with a fourth order Runge-Kutta path integration method [21] and 1 mm fixed step size. A continuous tensor field was estimated using trilinear interpolation of the tensor elements. Starting from the initial seed point, fiber paths were traced in both directions along the principal diffusion axis. One seed point per voxel was used. Path tracing proceeded until the FA fell below 0.15 or until the minimum angle between the current and previous path segments was larger than 30°. To limit the number of false positives, fibers that were anatomically implausible were identified visually and removed.

Structural connectivity*.* The density of fibers (number of fibers per unit area) connecting two regions *u* and *v* was computed as

fiber-density (*u,v*) = $\frac{2}{Su+ Sv}\sum_{f\in F(u,v)} \frac{1}{l(f)}$ (1)

where F(*u,v*) is a set of all the fibers connecting *u* and *v*, l(f) length of the individual fiber, Su and Sv are sizes of the ROIs [22]. The fiber density, a measure of structural connectivity, describes the fiber tracts interconnecting the regions of interest. Additionally, fiber mean fractional anisotropy (FA) was computed by averaging FA values along the tract.

**Discussion**

**Left hemisphere regions in the SN and CEN**

We were interested in examining right hemisphere ROIs because of a strong hypothesis motivated by our prior work suggesting that the right AI functions as a causal outflow hub [8]. Results from left hemisphere ROIs, however, would be useful in interpreting the right hemisphere results. Analysis of left hemisphere ROIs in the SN and CEN revealed that the left AI showed greater outflow compared to other nodes in children and adults, and in this way behaves in a similar way to the right AI. However, none of the group differences observed in the initial analysis for causal connectivity (rAI->rPPC) were present. This suggests to us that the left AI is not as strong a driver in the development of network dynamics as the right AI. Considering these left-hemisphere findings and our strong hypothesis about right AI, we primarily focused on right hemisphere ROIs in the main text.

**Sensory cortices and regions in the SN and CEN**

As reported in the main text, we found that, by age 9, the rAI node of the salience network is a major causal hub initiating control signals during problem solving. An important follow-up question is how sensory areas gain access to the rAI. To address this question, we conducted additional onset latency and MGCA analysis on a sensory ROI along with the five major nodes of SN and CEN. Specifically, a visual area ROI was defined by (1) selecting voxel with the highest *Z* score within the visual cluster on the functional activation map obtained by GLM analysis with task vs. rest contrast, (2) drawing a sphere with center as the seed-point and a radius of 8mm. ROI in the visual cluster was selected as the sensory area of interest as it is robustly activated by our visually presented problem solving task. Latency analysis of signals from the visual area (18,-102,-8; right lingual gyrus) revealed that the onset latency of this ROI did not differ significantly from rAI. The visual ROI showed early onset however it was not different from other regions. MGCA analysis revealed weak causal, but non-significant, interaction from the visual ROI to rAI in both children and adults. MDS analysis revealed similar results however it showed an additional weak causal interaction from rAI to the visual ROI. Similar results were observed when we used an 8mm sphere around the center of anatomical defined right lingual gyrus as the visual ROI. In sum, we observe early onset in sensory area and some indication of directionality of information flow. These results suggest that the rAI receives weak signals from the sensory cortices, which it further amplifies by exerting top-down control on regions involved in task-processing. The lack of robust latency differences between the visual area and rAI, and the weak/non-significant causal interactions could be because of the limits of temporal resolution of fMRI. Additional studies using better resolution data such as intracranial EEG are needed to more accurately examine this question.

**References**

1. Glover GH, Law CS (2001) Spiral-in/out BOLD fMRI for increased SNR and reduced susceptibility artifacts. Magn Reson Med 46: 515-522.

2. Glover GH, Lai S (1998) Self-navigated spiral fMRI: interleaved versus single-shot. Magnetic resonance in medicine : official journal of the Society of Magnetic Resonance in Medicine / Society of Magnetic Resonance in Medicine 39: 361-368.

3. Greicius MD, Srivastava G, Reiss AL, Menon V (2004) Default-mode network activity distinguishes Alzheimer's disease from healthy aging: evidence from functional MRI. Proc Natl Acad Sci U S A 101: 4637-4642.

4. Sterzer P, Kleinschmidt A (2007) A neural basis for inference in perceptual ambiguity. Proc Natl Acad Sci U S A 104: 323-328.

5. Menon RS, Luknowsky DC, Gati JS (1998) Mental chronometry using latency-resolved functional MRI. Proc Natl Acad Sci U S A 95: 10902-10907.

6. Formisano E, Goebel R (2003) Tracking cognitive processes with functional MRI mental chronometry. Curr Opin Neurobiol 13: 174-181.

7. Henson RN, Price CJ, Rugg MD, Turner R, Friston KJ (2002) Detecting latency differences in event-related BOLD responses: application to words versus nonwords and initial versus repeated face presentations. NeuroImage 15: 83-97.

8. Sridharan D, Levitin DJ, Menon V (2008) A critical role for the right fronto-insular cortex in switching between central-executive and default-mode networks. Proc Natl Acad Sci U S A 105: 12569-12574.

9. Jones DK (2004) The effect of gradient sampling schemes on measures derived from diffusion tensor MRI: a Monte Carlo study. Magn Reson Med 51: 807-815.

10. Bammer R, Auer M, Keeling SL, Augustin M, Stables LA, et al. (2002) Diffusion tensor imaging using single-shot SENSE-EPI. Magn Reson Med 48: 128-136.

11. Basser PJ (1995) Inferring microstructural features and the physiological state of tissues from diffusion-weighted images. NMR Biomed 8: 333-344.

12. Basser PJ, Pierpaoli C (1996) Microstructural and physiological features of tissues elucidated by quantitative-diffusion-tensor MRI. J Magn Reson B 111: 209-219.

13. Pajevic S, Aldroubi A, Basser PJ (2002) A continuous tensor field approximation of discrete DT-MRI data for extracting microstructural and architectural features of tissue. J Magn Reson 154: 85-100.

14. Alexander DC, Pierpaoli C, Basser PJ, Gee JC (2001) Spatial transformations of diffusion tensor magnetic resonance images. IEEE Trans Med Imaging 20: 1131-1139.

15. Tzourio-Mazoyer N, Landeau B, Papathanassiou D, Crivello F, Etard O, et al. (2002) Automated anatomical labeling of activations in SPM using a macroscopic anatomical parcellation of the MNI MRI single-subject brain. NeuroImage 15: 273-289.

16. Caspers S, Geyer S, Schleicher A, Mohlberg H, Amunts K, et al. (2006) The human inferior parietal cortex: cytoarchitectonic parcellation and interindividual variability. Neuroimage 33: 430-448.

17. Uddin LQ, Supekar K, Amin H, Rykhlevskaia E, Nguyen DA, et al. (2010) Dissociable connectivity within human angular gyrus and intraparietal sulcus: evidence from functional and structural connectivity. Cereb Cortex 20: 2636-2646.

18. Conturo TE, Lori NF, Cull TS, Akbudak E, Snyder AZ, et al. (1999) Tracking neuronal fiber pathways in the living human brain. Proc Natl Acad Sci U S A 96: 10422-10427.

19. Mori S, Crain BJ, Chacko VP, van Zijl PC (1999) Three-dimensional tracking of axonal projections in the brain by magnetic resonance imaging. Ann Neurol 45: 265-269.

20. Basser PJ, Pajevic S, Pierpaoli C, Duda J, Aldroubi A (2000) In vivo fiber tractography using DT-MRI data. Magn Reson Med 44: 625-632.

21. Press WH (2002) Numerical recipes in C++ : the art of scientific computing. Cambridge, UK ; New York: Cambridge University Press. xxvii, 1002 p. p.

22. Hagmann P, Cammoun L, Gigandet X, Meuli R, Honey CJ, et al. (2008) Mapping the structural core of human cerebral cortex. PLoS Biol 6: e159.
